# Shelf Life and Quality Study of Minced Tilapia with Nori and Hijiki Seaweeds as Natural Additives

**DOI:** 10.1155/2014/485287

**Published:** 2014-11-18

**Authors:** Ingridy Simone Ribeiro, Ligianne Din Shirahigue, Lia Ferraz de Arruda Sucasas, Lika Anbe, Pedro Gomes da Cruz, Cláudio Rosa Gallo, Solange Teresinha Carpes, Marcos José Marques, Marília Oetterer

**Affiliations:** ^1^Department of Plant Pathology, Federal University of Lavras (UFLA), P.O. Box 3037, 37200-000 Lavras, MG, Brazil; ^2^Department of Agri-Food Industry, Food and Nutrition, “Luiz de Queiroz” College of Agriculture, University of Sao Paulo (ESALQ/USP), Padua Dias Avenue 11, P.O. Box 9, 13418-900 Piracicaba, SP, Brazil; ^3^Embrapa Southeast Livestock, Km 234, P.O. Box 1, 13560-970 Sao Carlos, SP, Brazil; ^4^Department of Chemistry, Federal Technological University of Paraná (UTFPR), 85503-390 Pato Branco, PR, Brazil; ^5^Faculty of Pharmaceutical Sciences, Federal University of Alfenas (UNIFAL-MG), Gabriel Monteiro da Silva 700, 37130-000 Alfenas, MG, Brazil

## Abstract

The extraction of mechanically separated meat has emerged as an attractive process. However, it increases the incorporation of oxygen and, consequently, of flavors due to rancidity. Thus, preservatives must be added. The objective of this study was to evaluate the shelf life of minced tilapia to replace synthetic preservatives with Hijiki and Nori seaweeds extracts. The application of the extracts had no effect on the chemical composition of the minced tilapia. The seaweed extracts had inhibitory effect on total volatile base nitrogen. The minced tilapia complied with the microbiological standard set by Brazilin law. The panelists detected no differences in the rancid aroma and only minor differences were detected in the color of the products. It can be concluded that the minced tilapia with added seaweed extracts were within quality standards during frozen storage.

## 1. Introduction

Tilapia has emerged as one of the most cultivated and marketed species in Brazil. In 2000, manufacturing accounted for 18.4% of the total production in aquaculture. In 2004, it represented 38% of the total production at approximately 70,000 t [1]. Tilapia, currently produced at 132 000 t/year, is the national “flagship” aquaculture product because it represents 39% of the total farmed fish [2].

The increase in tilapia production over the years is due to the husbandry characteristics of the species and its adaptation to the climate of the country. It has considerable nutritional value, as it is rich in essential amino acids, unsaturated fatty acids, vitamins, and minerals, making it attractive to consumers that are increasingly concerned about maintaining a healthy and balanced diet.

The industrial processing of tilapia began in Brazil in the 1990s in western Paraná, focusing on only one product, frozen tilapia fillets. The yield of tilapia fillets was low, only 30–33%, consequently generating a large quantity of waste [3]. Traditionally, filleting waste and the waste from other fish preservation processes were used in the production of fishmeal for animal feed or discarded into the environment, creating pollution problems and economic losses. A simple alternative for the use of this waste has been the preparation of silage and its subsequent use in animal feed. However, the carcass remaining after filleting still had edible parts of good quality that could be used for human consumption [4, 5].

The production of mechanically separated meat (MSM) has emerged as an attractive process due to its high recovery of the meat; it creates basic raw material, and it is versatile for the development of coproducts. The application of MSM generates diversified products from minced meat such as surimi, kamaboko, hamburgers, and sausages [6].

Minced fish, as defined in the Codex, is a product obtained from one or more species of fish with similar sensory characteristics that have undergone the mechanical separation process, resulting in muscle tissue particles that are free from bones, skin, and viscera [6]. However, the mechanical separation process generates a product with a larger surface area, making it vulnerable to physical, chemical, and microbiological changes in a short amount of time. Therefore, the development of new product formulations to improve the technological quality of minced fish is necessary and may be achieved using ingredients such as preservatives [3].

The need to replace synthetic food additives such as butylated hydroxyanisole (BHA) and butylated hydroxytoluene (BHT), which induced changes in the lipid and enzyme levels in animals and potentially carcinogenic effects, has sparked interest in the study of substances with antioxidant activity [7]. Most of the recent research has been conducted on the replacement of chemical additives used in meat products including fish, with nonsynthetic products such as bark, grape seeds, raspberry seeds, and rosemary [8–14].

In Brazil, research on natural products of marine origin, particularly algae, began in the 1960s. Algae are grouped into two main categories: the microalgae, found in both benthic and littoral habitats and also throughout the ocean waters as phytoplankton and the macroalgae or seaweeds, which occupy the littoral zone, and can be classified as red (*Rhodophyta*), brown (*Phaeophyta*), or green (*Chlorophyta*), depending on their nutrient and chemical composition. The discovery of metabolites with biological activity from seaweed increased significantly in recent decades [15], with most of the available information referring to the chemical composition of these substances. Seaweed that is dried and stored for a long period does not undergo oxidation, even containing 30% total polyunsaturated fatty acids. Thus, the antioxidant mechanism of seaweed is a topic of research interest [7].

Specifically, the application of seaweeds as antioxidants in foods is a potential area of research [16]. This work is the first report on the use of seaweed extracts in products derived from fish.

The aim of this study was to replace the synthetic preservatives used in minced tilapia with seaweed extracts and to assess the shelf life (microbiological, physicochemical, and sensory attributes) of these new fish products.

## 2. Material and Methods

### 2.1. Seaweeds and Tilapia

The red seaweed Nori (*Porphyra tenera*) and the brown alga Hijiki (*Hijikia fusiformis*) were obtained in dehydrated form from suppliers of oriental food products prior to the preparation of ethanol extracts.

The total of 74.3 kg (135 fish) of Nile tilapia (*Oreochromis niloticus*) was obtained from Fish Palmares located in the region of Igaratá, State of Sao Paulo. The fish were collected in January 2011.

### 2.2. Preparation of Seaweed Extracts

Each of the dried seaweeds was milled and then weighed out (10 g) in an Erlenmeyer flask. Next, 100 mL of 60% (v/v) ethanol was added as the solvent extractor. The extraction was made by manual agitation. The flasks were sealed and stored in the dark at room temperature for 48 hours for the extraction of phenolic compounds. The extracts were filtered through qualitative filter paper and then stored in amber vials at 8°C [17].

### 2.3. Processing of MSM and Minced Tilapia

At the processing plant (ESALQ/USP), the whole fish were weighed, washed with water, scaled, eviscerated, and beheaded. After further washing, the fish were processed in a mechanical pulper, HIGH TECH brand model HT 100-C, to produce the MSM [17]. The MSM obtained was washed with water with a minimum free chlorine residual of 0.5 mg/L [2] at 10°C. A ratio of 3 L water to 1 kg of MSM was used for washing. Homogenization was then performed manually for 3 minutes, followed by a rest period of 3 min. The washed minced tilapia was stored in a nylon bag and centrifuged to drain off excess water. Prior to the addition of the Nori and Hijiki extracts to the minced tilapia, the gallic acid equivalents (GAE) of the extracts were determined by the Folin Ciocalteau method [18]. The concentrations of the extracts used were 25 and 50 g GAE/g of minced tilapia. A control sample was prepared under the same conditions without the addition of any seaweed extracts. A positive control was prepared by adding BHT at a concentration of 100 *μ*g BHT/g minced tilapia. Six treatments were investigated. The samples were packed in 300 g portions in polyethylene plastic bags, frozen, and stored at −18°C. The analyses were performed on the processing day and after 60, 120, and 180 days of storage.

### 2.4. Chemical Composition of Minced Tilapia

The moisture content of the samples was determined by measuring the weight loss of the samples placed in an oven heated to 105 ± 1°C until constant weights were achieved [19]. The protein content of the samples was quantified by determining the total nitrogen content using the Microkjeldahl method and a factor of 6.25 to convert the protein *N* values [20]. In determining the lipid content of the samples, the lipids were first extracted using the Soxhlet method with hexane as the solvent [19], followed by the preparation of the methyl esters of the fatty acids. The method is as follows: 2 mL of n-hexane and 0.2 mL of 2 mol equi/L methanolic KOH were added to 100 mg of sample in a tube and agitated for 30 sec; 3 mL of saturated sodium chloride was then added to the mixture, which was allowed to stand until the separation of the phases occurred. The upper phase was analyzed by gas chromatography. A Young Li Model 6000 series gas chromatograph with a flame ionization detector and a 2560 Supelco column (10 m × 0.25 mm × 0.2 mm) was used at the following operating conditions: a column temperature program that began with the maintenance of a 45°C temperature for 4 min, followed by a first ramp at 13°C/min to 175°C (27 min) and then a second ramp at 4°C/min to 215°C (35 min); an injector temperature at 220°C; a detector temperature at 220°C; and a sample split ratio of 1 : 50. A volume of 1 *μ*L of sample was injected into the chromatograph. The peaks were identified by comparing with the retention times of the patterns of the methyl esters with the separate components of the sample using the software YL Clarity [21]. The ash fraction of the samples was determined by incinerating the organic matter in a muffle furnace at 550°C until constant weights were achieved [19].

### 2.5. Total Volatile Base Nitrogen (TVB-N)

This analysis was performed using a version of the Brazilian Regulation number 20 distillation method [22] that was adjusted by Savay da Silva et al. [23]. Minced tilapia (50 g) were homogenized in 150 mL of trichloroacetic acid for the precipitation of protein nitrogen. The supernatant containing the volatile nitrogen was converted to alkalized steam that was received in a boric acid solution and the solution was titrated with 0.005 mol equi/L sulfuric acid in the presence of a mixed indicator.

### 2.6. Microbiological Evaluation

The microbiological analyses were performed using kits provided by ANVISA for products derived from fish (such as surimi) and fish products that are chilled or frozen. The Brazilian RDC no. 12 [24] recommends the counting of coagulase positive* Staphylococcus aureus*,* Salmonella* spp., and Coliforms at 45°C [25].

### 2.7. Sensory Analysis

Sensory analysis was performed by evaluating the intensity of the change in colour and flavour of minced tilapia as it became rancid. The evaluation used a hedonic scale in which a score of 1 represented an extreme intensity, while a score of 9 represented an absence of the sensory factor. The sensory tests were performed by 12 panelists (staff and students of ESALQ/USP), aged between 18 and 40 years. Of the six treatments developed, each panelist received four samples (4 treatments) that were selected randomly. The tests were conducted in individual booths under normal white light in the Sensory Analysis Laboratory of the Department of Agribusiness, Food and Nutrition at ESALQ-USP. Samples stored for 0, 60, 120, and 180 days were presented on ceramic plates and coded with 3 digit numbers that were randomly selected. This sensory evaluation was approved by the Ethics Committee on Research at the College of Agriculture “Luiz de Queiroz” (ESALQ/USP) under Protocol number 94, issued by the circular COET/127.

### 2.8. Statistical Analysis

The software SAS 9.2 was used for the statistical analysis of the data. A preliminary study was conducted to test the assumptions upon which the data are based. The data were tested for homogeneity of variance, the outliers were removed, and when appropriate remedial measures were taken. The data were analyzed using analysis of variance model appropriate and a test for the comparison of means (*P* < 0.05).

## 3. Results and Discussion 

The yield of flesh from fish may be influenced by several factors such as species, fish size, and the method used to remove the flesh. In the case of minced tilapia, the number of washes and method used to drain excess water may influence the final yield.

The yield of tilapia MSM was 34.92% of the whole fish. This value is similar to the others, 33.57% [26] and 33.76% [27], but lower than 46.90% [5].

The yield of MSM in relation to fish that had been gutted and beheaded was 80.04%, higher than the value obtained, 51.73% [26] but similar to 78.60% [5]. This value was the lowest that 87.43% [27].

The differences in yield may be due to two main reasons. The first is the differences in the configuration of the separation equipment. There may have been a wide variation in the adjustment of the blade assembly responsible for the mechanical separation of meat, which may decrease or increase the yield of MSM from beheaded and gutted fish. The second reason is the differences in the initial weight of the tilapia. Fish with greater weight, and therefore higher fat content, produce a greater yield of minced tilapia compared to MSM because the washing process removes sarcoplasmic proteins and fats [28].

For the various treatments, there were no statistically significant differences between the mean values of moisture (*P* = 0.2026), protein (*P* = 0.3585), lipids (*P* = 0.4183), and ash (*P* = 0.7175). In other words, the application of seaweed extracts and the synthetic antioxidant BHT did not influence the chemical composition of the minced tilapia in these experiments. For the interaction between treatment and storage time, there were also no statistically significant differences (moisture, *P* = 0.4314; protein, *P* = 0.0507; lipids, *P* = 0.9523; and ash, *P* = 0.4325) (Table 1).

With respect to storage time, there were no significant differences in the moisture and ash contents (*P* = 0.3353 and *P* = 0.3040, resp.). However, the protein and lipid values varied with time (Table 2).

The chemical compositions of the various minced tilapia differed due to the number of washes and the process of water removal [5].

The moisture values of the minced tilapia in this study, 81% on average, are close to those observed, 88.78% [5] and 80.69% [28]. Marengoni et al. [29] obtained a moisture value of 76.30% and Mélo et al. [30] obtained a moisture value of 72.75% for MSM tilapia.

An average protein content of 12.4% was obtained in this study. Other studies have reported values of 8.93% [5], 17.74% [29], 16.5% [28], and 14.29% [30].

The lipid values varied widely in this study, averaging 5.66%. The values obtained by other authors were 1.63% [5], 3.86% [29], 3.14% [28], and 9.26% [30].

The variation of the ash content was not as great as that of the other parameters. The values reported in previous studies are 0.46% [5], 0.88% [29], 0.50% [28], and 0.45% [30]. The average ash content obtained in this study was 0.46%.

The levels of total saturated fatty acids in the various treatments of minced tilapia ranged from 2.5 to 3.02 g/100 g. The total monounsaturated fatty acids content ranged from 1.79 to 2.51 g/100 g. For the polyunsaturated fatty acids, the values ranged between 0.32 and 0.85 (Table 3). The minced tilapia without any antioxidants had the lowest value of total monounsaturated fatty acids, suggesting that these fatty acids possibly degraded. The minced tilapia fish with added seaweed extracts had higher levels of monounsaturated fatty acids. The minced with Nori, 50 *μ*g GAE/g, had monounsaturated fatty acid levels close to that of the minced tilapia with BHT.

In the fraction of saturated fatty acids, palmitic acid (C16:0) was the predominant fatty acid with values ranging from 1.49 to 2.10 g/100 g. Stearic acid (C18:0) was the second most abundant fatty acid with values ranging from 0.46 to 0.69 g/100 g. Among the monounsaturated fatty acids, oleic acid (C18:1n9c) was the most abundant fatty acid with values ranging from 1.46 to 2.09 g/100 g. For the polyunsaturated fatty acids, linoleic acid (C18:2n6c) was the most abundant; it was estimated to be between 0.18 and 0.77 g/100 g. Similar values were obtained by Angelini [27] from tilapia Quenelle. Docosapentaenoic acid (DPA) and docosahexaenoic (DHA) were not detected as expected for tilapia products with low levels [4].

The evaluation of the total volatile nitrogenous bases (TVB-N) is a relatively simple and commonly used method to evaluate the freshness of fish. In Brazil, the Secretariat of Agricultural Protection, an office of the Ministry of Agriculture, Livestock and Supply, established a value of 30 mg N/100 g as the maximum TVB-N allowed for fresh fish, except for elasmobranchs [31]. Fish at an excellent state of freshness have TVB-N values in the range of 5–10 mg N/100 g of muscle; fish with a satisfactory level of freshness have values from 15 to 25 mg N/100 g. Before any changes occur in the fish, the levels of TVB-N may vary from 30 to 40 mg N/100 g. Decayed fish have TVB-N levels above 50 mg N/100 g [32].

Based on the TVB-N results, it was observed that among all the variables, treatment (*P* = 0.0248), storage time (*P* < 0.0001), and the interaction between treatment and time (*P* < 0.0001) were significantly different (Table 4).

For the variable treatment, the highest level of TVB-N was found in the minced tilapia without any antioxidants. Conversely, we observed the lowest levels of TVB-N in the treatments with the highest concentration of seaweed (Nori at 50 *μ*g GAE/g and Hijiki at 50 *μ*g GAE/g). This finding demonstrates the ability of seaweed extracts to exert a protective effect on minced tilapia. Increases in the levels of TVB-N were observed after 120 and 180 days of storage.

All the samples stored for various periods of time were within the parameters established by the Secretarial of Agricultural Protection [31].

Other studies on the content of the total volatile nitrogen bases in minced tilapia with seaweed extracts do not currently exist. Oliveira Filho et al. [33] used different proportions of minced tilapia in the formulation of sausages and stored the sausages for 40 days under refrigeration (0 ± 0.3°C). They analyzed the content of TVB-N in these sausages and found that there was no significant difference with storage time.

*Salmonella* was not detected in the minced tilapia samples. In adherence with the limits of detection of the method used, <10 CFU of* Staphylococcus* coagulase positive per gram of minced tilapia was detected. The other authors found similar results for quenelle, MSM, and pate tilapia, respectively [9, 27, 34]. Mélo et al. [30] also did not detect the presence of the coagulase positive* Staphylococcus* in MSM and bologna made from Nile tilapia.

Coliforms at 45°C are allowed by legislation. The minced tilapia samples showed no counts during the 180 days of frozen storage. Angelini [27] obtained a value of <3.0 MPN/g for storage of tilapia Quenelle. Kirschnik and Macedo-Viegas [5] did not find any fecal coliforms. Minozzo et al. [34] found values of 23 and 15 NMP/g for creamy pate and pasty tilapia, respectively.

The results of the treatments in this study were within the limits permitted by the technical regulation on the microbiological standards for food, as described in RDC no. 12 [24]. It provides ceiling values for coagulase positive* Staphylococcus* (5 × 10^3^ CFU/g), Coliforms at 45°C (103 MPN/g), and absence of* Salmonella* sp. in 25 g samples of products made from fish that are either chilled or frozen.

Satisfactory microbiological evaluation results can be achieved by cleaning and sanitizing of utensils and equipment and maintaining the hygiene of food handlers in addition to the use of chlorinated water (5 mg/mL) during food processing. Kirschnik and Macedo-Viegas [5] observed the psychrotrophic count in MSM subjected to washing and suggested that the washing process may exert a beneficial effect on reducing the microbial load.

The results of the sensory analysis showed significant effects of the added extracts on color (Table 5) with respect to treatment (*P* = 0.0304) and storage period (*P* = 0.0480). For the interaction between time and treatment, no significant difference (*P* = 0.8690) was found. It was observed that minced tilapia with Hijiki at a concentration of 50 *μ*g GAE/g showed the greatest change in color, as detected by the judges. The other treatments were not statistically different. For the time of storage, there was a gradual increase in color change during the 180 days of storage.

In the case of the attribute rancid aroma (Table 6), the storage time influenced the results (*P* = 0.0086). However, the treatment (*P* = 0.3374) and interaction between time and treatment (*P* = 0.5605) were not significantly different at 5%.

According to the sensory analysis, the panelists detected no differences in the “rancid aroma” of the minced tilapia samples. In other words, the applications of seaweed and synthetic antioxidant BHT in the minced tilapia had the statistically same “rancid aroma” in comparison with the control sample. This aroma can be disregarded; an opposite scale with scores of 1 (no detection) to 9 (high intensity) was presented to the panelists for use in this evaluation. A difference was detected with respect to only storage time; the samples stored for 180 days had the highest scores for “rancid aroma.”

Literature information on the sensory analysis of products with added marine seaweed is scarce. Wakame seaweed was used in different proportions in a new pasta formulation and evaluated its taste and appearance [35]. Formulations with lower proportions of seaweed, 5 and 10% (m/m), were the most accepted by the judges.

## 4. Conclusions

The application of seaweed extracts had no effect on the proximate composition of minced tilapia. The moisture, protein, lipid, and ash were, on average, 81%, 12.4%, 5.66%, and 0.46%, respectively. It had an inhibitory effect on the production TVB-N. In microbiological testing in accordance with the guidelines set by legislation, the samples were found to be microbiologically safe.* Salmonella* and Coliforms at 45°C were not detected and <10 CFU of* Staphylococcus* coagulase positive per gram of minced tilapia was detected. In the sensory analysis of the samples, panelists detected minor differences in color of the product but found no differences in the “rancid aroma.” In general, the treatments of the minced tilapia remained within the standards of quality during 180 days of frozen storage.

## Figures and Tables

**Table 1:** Proximate chemical composition of the minced tilapia.

Treatments of minced	Chemical composition (g/100 g)			
	Moisture	Protein	Lipids	Ash
Without antioxidant	82.03	12.08	5.12	0.44
BHT: 100 *µ*g/g	81.03	12.38	5.79	0.45
Nori: 25 *µ*g GAE^*^/g	81.41	11.54	5.60	0.47
Nori: 50 *µ*g GAE^*^/g	80.04	13.16	6.00	0.48
Hijiki: 25 *µ*g GAE^*^/g	80.93	12.69	5.72	0.45
Hijiki: 50 *µ*g GAE^*^/g	80.80	12.60	5.71	0.46

The standard errors are as follows: 0.732 for moisture; 0.649 for proteins; 0.372 for lipids; and 0.026 for ash. The mean values (*n* = 3) were obtained on a wet basis.

^*^GAE: gallic acid equivalents based in the phenolic compounds.

**Table 2:** Levels of proteins and lipids in the minced tilapia with respect to storage time.

Time (days)	Protein	Lipids
0	12.43^ab^	5.49^b^
60	12.73^a^	5.59^ab^
120	11.74^b^	6.04^a^
180	12.74^a^	5.54^b^

The standard error for the proteins was 0.265, while the standard error for the lipids was 0.152. The means (*n* = 3) that are followed by different letters differ by the Tukey test.

**Table 3:** Fatty acids in the minced tilapia (g/100 g).

Fatty acids	Treatments of minced (g/100 g)					
	T1	T2	T3	T4	T5	T6
C4	0.28	0.21	0.30	0.00	0.00	0.00
C14:0	0.21	0.24	0.18	0.28	0.29	0.27
C15:0	0.00	0.01	0.01	0.02	0.01	0.01
C16:0	1.49	1.73	1.54	2.04	2.10	2.02
C17:0	0.01	0.02	0.02	0.02	0.02	0.02
C18:0	0.51	0.46	0.69	0.57	0.57	0.52
C20:0	0.00	0.02	0.01	0.02	0.02	0.02

Total saturated fatty acids	2.50	2.72	2.77	2.96	3.02	2.87

C16:1	0.26	0.35	0.25	0.37	0.35	0.34
C18:1n9c	1.46	2.02	1.53	2.09	1.97	1.93
C20:1	0.07	0.11	0.08	0.00	0.00	0.00
C22:1n9	0.00	0.02	0.01	0.00	0.01	0.00

Total monounsaturated fatty acids	1.79	2.51	1.88	2.47	2.34	2.28

C18:2n6c	0.47	0.77	0.53	0.20	0.18	0.19
C18:3n3	0.02	0.05	0.03	0.16	0.14	0.14

Total polyunsaturated fatty acids	0.49	0.85	0.59	0.37	0.32	0.33

Trans fat	0.00	0.00	0.00	0.00	0.00	0.00

T1: without antioxidant; T2: BHT: 100 *µ*g/g; T3: Nori: 25 *µ*g GAE^*^/g; T4: Nori: 50 *µ*g GAE^*^/g; T5: Hijiki: 25 *µ*g GAE^*^/g; T6: Hijiki: 50 *µ*g GAE^*^/g.

^*^GAE: gallic acid equivalents based in the phenolic compounds.

**Table 4:** Total volatile base nitrogen (mg n/100 g) of minced tilapia stored for 180 days.

Treatments	Days of storage				*P* = 0.0248
	0	60	120	180	
Without antioxidant	4.14	3.72	4.62	6.55	6.40^a^
BHT: 100 *µ*g/g	2.50	3.34	7.75	6.03	4.91^ab^
Nori: 25 *µ*g GAE^*^/g	3.70	3.34	4.31	6.81	4.54^ab^
Nori: 50 *µ*g GAE^*^/g	1.55	3.04	5.17	6.12	3.97^b^
Hijiki: 25 *µ*g GAE^*^/g	2.84	2.83	7.75	6.55	4.99^ab^
Hijiki: 50 *µ*g GAE^*^/g	3.79	2.14	5.17	6.89	4.50^b^

*P* < 0.0001	3.08^B^	3.07^B^	6.89^A^	6.49^A^	

The standard errors are as follows: 0.34 for treatment, 0.22 for time, and 0.53 for the interaction between treatment and time. The means (*n* = 3) that are followed by capital letters in the rows and lowercase letters in the columns do not differ by the Tukey test.

^*^GAE: gallic acid equivalents based in the phenolic compounds.

**Table 5:** Sensory analysis for the color of minced tilapia.

Treatments	Days of storage				*P* = 0.0304
	0	60	120	180	
Without antioxidant	1.62	1.62	1.75	1.80	1.70^b^
BHT: 100 *µ*g/g	1.12	1.62	1.37	2.20	1.58^b^
Nori: 25 *µ*g GAE^*^/g	1.85	1.47	1.75	2.00	1.77^ab^
Nori: 50 *µ*g GAE^*^/g	2.40	2.30	2.62	3.00	2.58^ab^
Hijiki: 25 *µ*g GAE^*^/g	1.77	2.62	2.50	2.65	2.38^ab^
Hijiki: 50 *µ*g GAE^*^/g	3.10	3.07	4.12	4.00	3.57^a^

*P* = 0.0480	1.98^B^	2.12^AB^	2.35^AB^	2.61^A^	

The standard errors for treatment and time are 0.32 and 0.18, respectively. The means (*n* = 3) that are followed by capital letters in the rows and lowercase letters do not differ by the Tukey test.

^*^GAE: gallic acid equivalents based in the phenolic compounds.

**Table 6:** Sensory analysis for the rancid aroma of minced tilapia.

Treatments	Days of storage			
	0	60	120	180
Without antioxidante	1.87	2.00	2.12	2.15
BHT: 100 *µ*g/g	1.37	2.37	1.75	2.35
Nori: 25 *µ*g GAE^*^/g	1.42	1.12	2.12	2.65
Nori: 50 *µ*g GAE^*^/g	2.07	1.82	1.87	3.65
Hijiki: 25 *µ*g GAE^*^/g	1.60	2.05	2.12	2.15
Hijiki: 50 *µ*g GAE^*^/g	1.75	2.30	3.00	3.00

*P* = 0.0086	1.68^B^	1.95^AB^	2.17^AB^	2.65^A^

The standard error for storage time is 0.18. The means (*n* = 3) that are followed by capital letters in the rows do not differ by the Tukey test.

^*^GAE: gallic acid equivalents based in the phenolic compounds.
